# The cumulative live birth rates of 18 593 women with progestin-primed ovarian stimulation-related protocols and frozen-thawed transfer cycles

**DOI:** 10.1093/hropen/hoad051

**Published:** 2023-12-21

**Authors:** Yunhan Nie, Wenya Guo, Xi Shen, Yating Xie, Yuqi Zeng, Hongyuan Gao, Yali Liu, Li Wang

**Affiliations:** Department of Assisted Reproduction, Shanghai Ninth People’s Hospital, Shanghai Jiao Tong University School of Medicine, Shanghai, PR China; Department of Assisted Reproduction, Shanghai Ninth People’s Hospital, Shanghai Jiao Tong University School of Medicine, Shanghai, PR China; Department of Assisted Reproduction, Shanghai Ninth People’s Hospital, Shanghai Jiao Tong University School of Medicine, Shanghai, PR China; Department of Assisted Reproduction, Shanghai Ninth People’s Hospital, Shanghai Jiao Tong University School of Medicine, Shanghai, PR China; Department of Assisted Reproduction, Shanghai Ninth People’s Hospital, Shanghai Jiao Tong University School of Medicine, Shanghai, PR China; Department of Assisted Reproduction, Shanghai Ninth People’s Hospital, Shanghai Jiao Tong University School of Medicine, Shanghai, PR China; Department of Assisted Reproduction, Shanghai Ninth People’s Hospital, Shanghai Jiao Tong University School of Medicine, Shanghai, PR China; Department of Assisted Reproduction, Shanghai Ninth People’s Hospital, Shanghai Jiao Tong University School of Medicine, Shanghai, PR China

**Keywords:** cumulative live birth rate, progestin-primed ovarian stimulation-related protocol, frozen embryo transfer, ART, IVF, ICSI

## Abstract

**STUDY QUESTION:**

What are the odds of achieving pregnancy when adopting progestin-primed ovarian stimulation (PPOS)-related protocols combined with repetitive frozen-thawed transfer (FET) cycles in patients with different clinical characteristics?

**SUMMARY ANSWER:**

The cumulative live birth rates (CLBRs) of women undergoing different PPOS-related protocols can be significantly and consistently enhanced within six FET cycles when the female age is <40 years (or even <45 years) and when >5 oocytes are retrieved, regardless of antral follicle count (AFC).

**WHAT IS KNOWN ALREADY:**

There have been numerous studies on the live birth rate of the first FET cycle in patients with PPOS-related protocols. These studies have focused mainly on comparing pregnancy outcomes with those of other stimulation protocols. However, owing to the unique features of the PPOS-related strategy, such as its flexible timing of oocyte retrieval and repeated transfer of frozen embryos, studies using the CLBR as an overall indicator of success and investigating which types of patients would benefit from this protocol are lacking.

**STUDY DESIGN, SIZE, DURATION:**

This retrospective cohort study included 18 593 women who underwent PPOS-related protocols (dydrogesterone + hMG, medroxyprogesterone acetate + hMG, micronized progesterone + hMG treatment, and luteal-phase ovarian stimulation protocol) from 1 March 2011 to 31 September 2022 in our centre.

**PARTICIPANTS/MATERIALS, SETTING, METHODS:**

The population was categorized by female age, number of oocytes retrieved, and AFC in the analysis of CLBR within six FET cycles. The age groups (Groups 1–5, respectively) were <30, 30–34, 35–39, 40–44, and ≥45 years. The number of oocytes retrieved was grouped as 1–5, 6–10, 11–15, 16–20, and >20. AFC was grouped as <5, 5–10, 11–15, and >15. The Kaplan–Meier analysis (optimistic method), which hypothesized that patients who did not continue treatment had the same chance of achieving a live birth as those who continued, and the competing risk method (conservative method) which hypothesized they had no chance of achieving a live birth, were applied. In further analyses, the Cox model and Fine–Gray model were adopted: the former corresponds to the optimistic scenario, and the latter corresponds to the pessimistic scenario.

**MAIN RESULTS AND THE ROLE OF CHANCE:**

CLBR had a declining trend with female age over six FET cycles (Groups 1–5, respectively: optimistic: 96.9%, 96.6%, 91.4%, 67.3%, and 11.7%; conservative: 87.3%, 85.0%, 74.0%, 41.3%, and 7.5%), requiring more FET cycles to achieve a success rate of at least 50% (Groups 1–5, respectively: optimistic: 2, 2, 2, 4, and >6 cycles; conservative: 2, 2, 2, >,6 and >6 cycles). CLBR showed an increasing trend with the number of oocytes retrieved (Groups 1–5, respectively: optimistic: 93.8%, 94.3%, 95.8%, 96.0%, and 95.6%; conservative: 66.2%, 78.3%, 85.6%, 88.9%, and 91.0%). All groups needed the same number of FET cycles to achieve a success rate of at least 50% (Groups 1–5, respectively: optimistic: 2, 2, 2, 2, and 2 cycles; conservative: 2, 2, 2, 2, and 2 cycles). Furthermore, the CLBR within six FET cycles had an increasing trend with AFC number (Groups 1–4, respectively: optimistic: 89.2%, 94.8%, 95.9%, and 96.3%; conservative: 67.4%, 78.2%, 83.9%, and 88.1%), with all four groups achieving a success rate of at least 50% by the second FET cycle.

**LIMITATIONS, REASONS FOR CAUTION:**

The current research is limited by its retrospective design and single-centre nature, which may restrict the generalizability of our findings.

**WIDER IMPLICATIONS OF THE FINDINGS:**

This work describes two models (the Kaplan–Meier analysis and the competing risk method) to evaluate the clinical outcome of patients using PPOS-related protocols, which are especially useful for patients of advanced age or those with diminished ovarian reserve. Our findings encourage patients below 45 years old, especially younger than 40 years, and patients with lower AFCs and fewer retrieved oocytes to try this new protocol. Moreover, this study demonstrates the degree of improvement in the CLBR within six FET cycles for patients with different clinical characteristics, providing a valuable point of reference to determine whether to continue ART after a transfer failure.

**STUDY FUNDING/COMPETING INTEREST(S):**

The study was supported by grants from the National Natural Science Foundation of China (82071603 to L.W., 82001502 to Y.L.). There are no conflicts of interest to declare.

**TRIAL REGISTRATION NUMBER:**

N/A.

WHAT DOES THIS MEAN FOR PATIENTS? Controlled ovarian stimulation is a crucial part of IVF. During this stimulation, the rise in oestradiol level can sometimes trigger an early surge of luteinizing hormone (LH), resulting in early release of oocytes (eggs) that will compromise the collection of a full set of mature oocytes. To help avoid this, protocols have been developed to block this early LH surge. One of these new types of stimulation regime, called progestin-primed ovarian stimulation (PPOS), aims to prevent the early LH surge in a more patient-friendly way, through the use of progesterone. Compared to previous protocols, it is better at preventing early ovulation and achieves similar results in terms of collecting oocytes and achieving pregnancy. As this new oocyte stimulation programme becomes more widely accepted and embryo freezing techniques continue to develop, it is being applied in more situations and yielding more embryos. Even so, studies using this protocol to analyze live birth rate after the first frozen embryo transfer cycle or the cumulative live birth rate (number of births after transfer of all embryos created from one egg collection) from the initial IVF cycle, still leave many questions unanswered. In this study, we used the cumulative live birth rate per woman to assess the overall pregnancy outcomes using this PPOS protocol. Our results showed that PPOS improves the cumulative live birth rate but this improvement varies depending upon a woman’s age and body mass index, for example, as the number of frozen embryo transfer cycles carried out increases. Based on these findings, women who are using PPOS in the clinic can decide (based on their own clinical characteristics) whether to continue with assisted reproductive technology treatment if their first embryo transfer fails.

## Introduction

The progestin-primed ovarian stimulation (PPOS)-related protocols include the initial luteal-phase ovarian stimulation (LPS) protocol (Kuang *et al.*, 2014) and the classical PPOS protocol. This scheme underwent several stages of development before its proposal and improvement. In our centre, we first proposed LPS (Kuang *et al.*, 2014) in 2014, which utilizes letrozole and hMG to stimulate early follicle growth. Later, we explored the use of hMG along with exogenous progesterones, such as medroxyprogesterone acetate (MPA) (Kuang *et al.*, 2015), micronized progesterone (Wang *et al.*, 2018), and dydrogesterone (DYG) (Yu *et al.*, 2018), for ovarian stimulation. These methods yielded similar results in terms of oocyte retrieval and pregnancy rates. Compared to conventional approaches, such as GnRH agonists and antagonists, the PPOS-related regimen has the advantage of effectively preventing an early LH surge while achieving comparable outcomes in oocyte retrieval (Massin, 2017) and pregnancy (Kuang *et al.*, 2015).

PPOS-related protocols are not combined with fresh embryo transfer (ET) since it is widely recognized that high blood progesterone on the day of hCG injection is associated with poor endometrial receptivity, which reduces the likelihood of maintaining a pregnancy (Bosch *et al.*, 2010; Venetis *et al.*, 2013). However, the implementation of the ‘freeze-all’ strategy can alleviate restrictions associated with the potential adverse effects of progesterone (Massin, 2017). Furthermore, by utilizing the advanced vitrification cryopreservation method that employs high concentrations of cryoprotectant agents and ultrafast cooling and warming rates, embryo quality can be maintained, leading to improved pregnancy outcomes (Wong *et al.*, 2014). This allows for safe freezing of embryos obtained from ovulation and insemination for multiple transfers. As demonstrated by Qin *et al*. (2016), PPOS-related protocols allow for high flexibility in the starting phase of ovarian stimulation, letting it begin in the early follicular phase, the late follicular phase, or the luteal phase. By combining these two methods, oocytes can be collected within flexible time frames, and frozen embryos can be transferred repetitively. These features enable infertile individuals to increase their cumulative live birth rate (CLBR) and undergo as many ART procedures as possible within a shorter timeframe to achieve a healthy pregnancy.

CLBR is traditionally defined as the percentage of cycles started or oocyte aspirations performed that result in at least one live birth, including all fresh ETs and/or frozen embryo transfers (FETs) until either a live birth occurs or all embryos are used, whichever happens first (Saket *et al.*, 2021). CLBR better reflects a person’s overall live birth success than the results of just the first cycle or a single oocyte extraction and ET cycle. This is why it is considered a more relevant variable and objective statistical indicator of the clinical outcome of ART. It not only allows for the inclusion of patients with incomplete ETs after one ovulation aspiration cycle but also facilitates the observation of the CLBR of each ET cycle. CLBR has been used as an assessment index for pregnancy outcomes in several studies. For example, Chen *et al.* (2022) compared the effectiveness of PPOS and GnRH antagonist pregnancy strategies using CLBR. In a randomized controlled trial, CLBR was a key indicator to measure how well the reproductive functions of obese women improved after losing weight (Kluge *et al.*, 2019). In a systematic review, both CLBR and the fresh live birth rate were used to compare pregnancy outcomes between mild and conventional stimulation methods (Montoya-Botero *et al.*, 2021).

Owing to the flexible oocyte retrieval and repetitive ET features of PPOS-related protocols, it is likely that prior research studies that focus primarily on live birth rates from the first FET cycle may be biased. Consequently, data on CLBR is crucial and can provide a key reference value for PPOS-related protocols. This study, which investigated the pregnancy outcomes of 18 593 infertile women following ovulatory aspirations with PPOS-related protocols and six FET transfers, can serve as a benchmark for the overall clinical outcome of PPOS-related protocols.

## Materials and methods

### Study design and patients

This is a large retrospective population-based study that utilizes the clinical database of the Department of Assisted Reproduction of the Ninth People’s Hospital affiliated with Shanghai Jiao Tong University School of Medicine.

We enrolled 18 593 patients who underwent only PPOS-related protocols (Kuang *et al.*, 2015), which included ovarian stimulation approaches such as letrozole + hMG (LPS) (Chen *et al.*, 2015), MPA + hMG (Kuang *et al.*, 2015), micronized progesterone + hMG treatment (Wang *et al.*, 2018), and DYG + hMG (Yu *et al.*, 2018) from March 2011 to September 2022. We followed up their live birth status through July 2023. Among the 32 128 patients who underwent PPOS-related protocols, 18 593 adhered exclusively to these protocols from start to finish, while the remaining 13 535 received other controlled ovarian hyperstimulation protocols and were excluded from the main analysis.

### PPOS-related protocol and oocyte retrieval

PPOS-related protocols were utilized for all patients. They effectively inhibit the positive feedback effects induced by oestradiol (E2) through the use of endogenous or exogenous progesterone (Kuang *et al.*, 2015). The detailed treatment of ovarian stimulation has been reported in our previous research (Wang *et al.*, 2018; Yu *et al.*, 2018). Briefly, patients received daily ovarian stimulation from menstrual cycle day 3 (MC3) to the trigger day with injections of hMG (150–225 IU; Anhui Fengyuan Pharmaceutical Co., Anhui, China) plus oral DYG (Duphaston; 20 mg/day; Abbott Biologicals B.V., Hoofddorp, Netherlands) in the DYG + hMG treatment (Yu *et al.*, 2018); oral MPA (10 mg/day; Shanghai Xinyi Pharmaceutical Co., Shanghai, China) in the MPA + hMG treatment (Kuang *et al.*, 2015); or oral Utrogestan (100 mg/day; Laboratories Besins International, Paris, France) in the micronized progesterone + hMG treatment (Wang *et al.*, 2018). In the LPS treatment, 225 IU hMG was injected 1–3 days after ovulation, while letrozole (2.5 mg/day; Jiangsu Hengrui Medicine Co. Ltd, Jiangsu, China) was administered (Chen *et al.*, 2015).

The final stage of oocyte maturation was triggered when three dominant follicles reached a diameter of 18 mm under triptorelin stimulation (0.1–0.2 mg; decapeptyl, Ferring Pharmaceuticals, Guangdong, China) or i.m. injections of hCG (1000–5000 IU; Lizhu Pharmaceutical Trading Co., Zhuhai, China) or cotriggered with s.c. triptorelin (0.1–0.2 mg) and i.m. injections of hCG (1000–5000 IU). Transvaginal ultrasound-guided oocyte retrieval was conducted 34–36 h after the trigger in the PPOS strategy (Kuang *et al.*, 2015; Wang *et al.*, 2018; Yu *et al.*, 2018; Shen *et al.*, 2019) and at 32–36 h in the LPS protocol (Kuang *et al.*, 2014). All follicles with a diameter greater than 10 mm were aspirated.

### Laboratory protocols and frozen embryo transfer

IVF or ICSI was used to fertilize the oocytes. On fertilization day 3, the embryos were assessed and categorized using the Cummins criteria (Cummins *et al.*, 1986), which accounted for the embryo blastomere count, regularity, and degree of embryonic fragmentation. If the patients had fewer than six good-quality cleavage-stage embryos, all good-quality cleavage-stage embryos were cryopreserved in our centre. Conversely, if there were more than six, the surplus good-quality embryos underwent blastocyst culture. Embryos of inferior quality were placed in extended culture until reaching the blastocyst stage before being cryopreserved. Blastocyst morphology was assessed based on Gardner and Schoolcraft’s classification (Gardner and Schoolcraft, 1999) according to the blastocoel, inner cell mass, and trophectoderm. Only morphologically good blastocysts were cryopreserved on day 5 or day 6.

All of the included patients underwent the freeze-all strategy because the PPOS-related procedure alters the endometrial environment during the oocyte retrieval cycle. The endometrium was prepared according to each patient’s circumstances, as mentioned previously. For women with a history of irregular menstrual cycles, mild stimulation with letrozole was initially recommended. Natural cycle and hMG late stimulation cycle were used for patients with regular menstrual cycles. Hormone replacement therapy was conducted for those with a history of a thin endometrium (≤6 mm) or who failed to become pregnant with mild stimulation cycles, natural cycles or hMG late stimulation cycles.

In the mild stimulation cycle, women were administered letrozole 2.5/5 mg for 5 days starting on MC3. For patients with natural cycles, monitoring of follicular development typically began on MC12 or when the urine LH strip paper was positive. If the dominant follicle was less than 10 mm on MC12, an hMG late stimulation cycle was suggested. When the dominant follicle reached a diameter of ≥17 mm and the endometrial lining was >8 mm, with E2 > 150 pg/ml and progesterone <1 ng/ml, a bolus of urinary hCG (5000 IU) was injected to trigger ovulation. Progesterone was started 2 or 3 days later, followed by day 3 ET after 4 or 5 days, or blastocyst transfer after 6 or 7 days, via abdominal ultrasound guidance. At most, two embryos were transferred at one time.

### Study variables and outcome measurement

First, the basic characteristics of women undergoing the PPOS-related protocol were examined. The cause of infertility was categorized by tubal factors, male factors (testicular and post-testicular deficiencies), ovulatory dysfunction (e.g. hypogonadotropic hypogonadism, premature ovarian insufficiency, PCOS), endometrial factors (including endometriosis, adenomyosis, uterine fibroids, endometrial polyps, uterine adhesions), mixed causes, and unexplained factors of infertility.

The IVF/ICSI and FET cycles were divided into five groups according to the woman’s age, namely, <30, 30–34, 35–39, 40–44, and ≥45 years, to describe the IVF/ICSI and FET cycle characteristics in detail. After the description of the fundamental characteristics, the CLBRs within six FET cycles were explored in female patients according to age, number of oocytes retrieved and antral follicle count (AFC). The patient population was categorized by age, with the following distribution: <30 (35.14%), 30–34 (42.04%), 35–39 (18.22%), 40–44 (4.11%), and ≥45 (0.48%); the number of oocytes retrieved was grouped as 1–5 (15.54%), 6–10 (29.02%), 11–15 (25.06%), 16–20 (15.25%), and >20 (15.12%); and the AFC grouping was <5 (8.90%), 5–10 (32.16%), 11–15 (27.80%), and >15 (31.14%). All groups were based on data at the time of each participant’s first oocyte retrieval.

The main outcome of this study was the CLBR per woman, where a live birth was defined as at least one liveborn baby at ≥20 weeks of gestation resulting from an ET cycle, which included all oocyte retrieval cycles and subsequent FET cycles. Therefore, according to this definition, delivery of more than one baby was counted only once, and each patient’s live births after the initial one was not considered.

### Statistical analysis

CLBR curves were plotted against the number of FET cycles (Leijdekkers *et al.*, 2019) and are presented based on two methods (Malizia *et al.*, 2009). The Kaplan–Meier analysis (optimistic method) suggested that patients who did not resume treatment had the same chance of achieving a live birth as those who did. The competing risk method (conservative method) hypothesized that patients who did not continue treatment had no chance of achieving a live birth. The competing factor in the competing risk method was patients who did not continue treatment, and the actual ‘raw’ curve lies somewhere between optimism and pessimism. Pairwise comparisons between groups were conducted by an adjusted pairwise log-rank test.

To further investigate how female age, number of oocytes retrieved, and AFC influenced CLBR, the Cox model and Fine–Gray model were adopted; the former corresponds to the optimistic scenario, and the latter corresponds to the pessimistic scenario. To reduce the errors caused by possible confounding, single-variable analysis through the Cox model was applied to filter this out. The key factors that we adjusted for included duration of infertility, female BMI, male age, infertility type, infertility reason, PCOS, OPU times, FET times, total oocyte number, total embryo number, number of embryos transferred, number of good-quality embryos transferred, and the patient’s first OPU year (referring to the year when the patient undergoes her first OPU cycle). We did not include smoking habit as a factor since fewer than 5% of Chinese women smoke (Wang *et al.*, 2019) and even fewer of childbearing age do. Next, we conducted multivariable analysis including the three principal factors and the variables mentioned above. The results were recorded as adjusted hazard ratios (aHRs) with their 95% CIs. *P* < 0.05 was considered significant in the univariable or multivariable regression models.

All statistical analyses were conducted using R software (version 4.2.2, R Foundation for Statistical Computing, Vienna, Austria), IBM SPSS Statistics (version 26.0.0, IBM Corp, Armonk, NY, USA), and GraphPad Prism (version 8.0.2, Dotmatics, Boston, MA, USA). Categorical variables are expressed as quantities (%) and were tested by the chi-square test or Fisher’s exact test. Continuous variables are presented as the mean ± SD and were tested by one-way ANOVA. *P* < 0.05 was statistically significant. The Bonferroni correction was applied to prevent data from incorrectly appearing to be statistically significant, by making an adjustment during comparison testing.

### Ethical approval

Before ART, all participants in our centre provided written informed consent. This retrospective study utilized an anonymous database, ensuring that no personally identifiable information was accessible to the researchers. The Institutional Ethics Committee of Shanghai Ninth People’s Hospital granted access to the database (SH9H-2018-T57-1).

## Results

**Figure 1.**
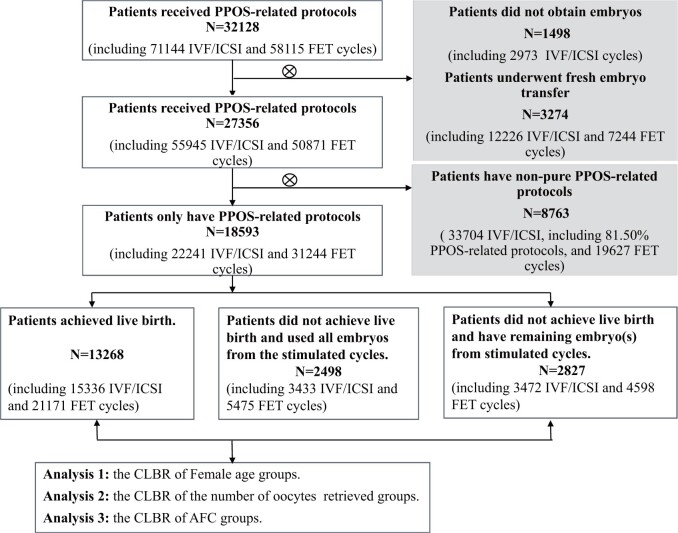
**Flowchart illustrating the study population, including the criteria for inclusion and exclusion.** Patients with pure PPOS-related protocols were included in the primary analysis, while patients with non-pure PPOS-related protocols were included in the supplementary analysis. Patients enrolled in pure PPOS-related protocols exclusively underwent ovarian stimulation cycles utilizing dydrogesterone + hMG, medroxyprogesterone acetate + hMG, micronized progesterone + hMG treatment, or luteal-phase ovarian stimulation protocol. Patients enrolled in non-pure PPOS-related protocols underwent ovarian stimulation cycles involving alternative stimulation protocols, not limited to these PPOS-related ovarian stimulation protocols. PPOS, progestin-primed ovarian stimulation; FET, frozen-thawed transfer; CLBR, cumulative live birth rate; AFC, antral follicle count.

The patient selection and exclusion criteria are presented in the flowchart in Fig. 1. A total of 18 593 female patients who underwent purely PPOS-related protocols during our observation period were included, comprising 22 241 IVF/ICSI cycles and 31 244 FET cycles. The analysis focused on these patients while excluding the 8763 patients who underwent non-purely PPOS-related protocols during our observation period, but they were retained for supplementary analysis to compare the CLBR between the two groups.

### The baseline characteristics of the study population

The baseline characteristics of the population are presented in Table 1. A total of 18 593 women underwent 22 241 oocyte pick-up cycles and 31 244 FET cycles. The female age group with the largest population was the 30–34 years group (7817, 42.04%). The most numerous female BMI group was the 18.5–23.99 kg/m^2^ (12 493, 67.19%) group. The majority of women experienced primary infertility (10 215, 54.94%).

**Table 1.** Baseline characteristics of 18 593 female patients under the progestin-primed ovarian stimulation-related protocol.

Demographic characteristics	N (%)
**Number of women**	18 593
**Female age (years)**	
<30	6533 (35.14%)
30–34	7817 (42.04%)
35–39	3388 (18.22%)
40–44	765 (4.11%)
≥45	90 (0.48%)
**Female BMI (kg/m^2^)**	
<18.5	2098 (11.28%)
18.5–23.99	12 493 (67.19%)
24.0–27.99	3175 (17.08%)
≥28.0	827 (4.45%)
**Infertility type**	
Primary infertility	10 215 (54.94%)
Secondary infertility	8378 (45.06%)
**Cause of infertility**	
Tubal	7888 (42.42%)
Male cause	2321 (12.48%)
Ovulatory	1030 (5.54%)
Endometrium factor	602 (3.24%)
Mixed causes	4758 (25.59%)
Unexplained	1994 (10.72%)
**Duration of infertility (years)**	3.16
**Number of oocyte pick-up cycles**	22 241
IVF	13 080 (58.81%)
ICSI	6009 (27.02%)
IVF + ICSI	3152 (14.17%)
**Number of women having different number of oocyte pick-up cycles**	
1	15 683 (84.34%)
2	2337 (12.57%)
3	453 (2.44%)
4	95 (0.51%)
≥5	25 (0.14%)
**Number of FET cycles**	31 244
**Number of women having different FET cycles and mean duration (month)**	
1	9851 (52.98%); 3.35
2	5310 (28.56%); 10.74
3	2103 (11.31%); 18.00
4	695 (3.74%); 23.71
5	225 (1.21%); 30.67
≥6	126 (0.68%); 41.27
People with no FET after OPU	283 (1.52%)

FET, frozen embryo transfer; OPU, oocyte pick-up; mean duration, the average duration from the initial oocyte collection to the corresponding transfer cycle.

Among the causes of infertility, tubal factors were the most frequent cause of disease (7888, 42.42%), followed by mixed causes, male causes, ovulatory factors, endometrial factors, and unexplained factors. Among the oocyte pick-up cycles, IVF was performed in 58.81% of cases. Some 84.34% of the patients underwent a single oocyte pick-up cycle, 12.57% underwent two cycles, and a small proportion (3.09%) underwent three or more cycles. Over half of the patients (52.98%) underwent a single FET cycle, with an average interval of 3.35 months from the initial oocyte collection to the first transfer cycle. There was an average interval of approximately 7 months between consecutive transfers (Table 1).

The baseline characteristics of the female patients under non-pure PPOS-related protocols were also delineated (Supplementary Table S1). This cohort was distinguished from the previous patients by its higher prevalence in the older age groups (group 35–39: 25.79% versus 18.22%, group 40–44: 16.41% versus 4.11%, group 45: 3.74% versus 0.48%). Moreover, these individuals underwent a higher number of oocyte retrieval cycles: 25.60% of patients experienced more than five cycles. The proportion of patients undergoing only one FET cycle was significantly lower, at only 32.37%.

### Characteristics of IVF/ICSI and FET cycles in different age groups

To better understand the cycle characteristics, we further divided the cycles into five groups based on female age: Group 1 (<30 years), Group 2 (30–34 years), Group 3 (35–39 years), Group 4 (40–44 years), and Group 5 (≥45 years). Differences between groups are listed in Table 2.

**Table 2.** IVF/ICSI and FET cycle characteristics within five different age groups.

	<30	30–34	35–39	40–44	≥45	*P-*value
**IVF/ICSI cycle characteristics**						
**Number of oocyte pick-up cycles**	7194	9199	4363	1274	211	<0.001
IVF	4134 (57.46%)^a^	5405 (58.76%)^b^	2616 (59.96%)^c^	780 (61.22%)^d^	145 (68.72%)^e^	
ICSI	1960 (27.24%)^a^	2324 (25.26%)^b^	1223 (28.03%)^c^	440 (34.54%)^d^	62 (29.38%)^e^	
IVF + ICSI	1100 (15.29%)^a^	1470 (15.98%)^b^	524 (12.01%)^c^	54 (4.24%)^d^	4 (1.90%)^e^	
**Type of PPOS protocol**						<0.001
hMG + MPA	5364 (74.56)^a^	7043 (76.56)^b^	3365 (77.13)^b^	1000 (78.49)^b^	184 (87.20)^c^	
hMG + Utrogestan	721 (10.02)^a^	796 (8.65)^b^	350 (8.02)^bc^	104 (8.16)^a,b,c^	6 (2.84)^c^	
hMG + DYG	731 (10.16)^a^	832 (9.04)^a^	406 (9.31)^a^	104 (8.16)^a^	10 (4.74)^a^	
Luteal-phase ovarian stimulation	378 (5.25)^a^	528 (5.74)^a^	242 (5.55)^a^	66 (5.18)^a^	11 (5.21)^a^	
**AFC, mean (SD)**	14.17 (7.10)^a^	12.16 (6.52)^b^	9.73 (5.72)^c^	6.68 (4.24)^d^	4.61 (3.11)^e^	<0.001
**Oocytes retrieved, mean (SD)**	14.73 (8.25)^a^	12.14 (7.49)^b^	9.14 (6.15)^c^	5.76 (4.50)^d^	3.92 (3.08)^e^	<0.001
**Fertilized oocytes, mean (SD)**	11.07 (6.48)^a^	9.19 (5.84)^b^	7.05 (4.82)^c^	4.59 (3.62)^d^	3.11 (2.57)^e^	<0.001
**Total embryos, mean (SD)**	10.09 (5.93)^a^	8.44 (5.33)^b^	6.52 (4.40)^c^	4.35 (3.24)^d^	3.07 (2.32)^e^	<0.001
**High-quality embryos**						<0.001
Cleavage embryos, mean (SD)	4.12 (2.40)^a^	3.61 (2.30)^b^	3.04 (2.09)^c^	2.26 (1.80)^d^	1.53 (1.37)^e^	
Blastocyst embryos, mean (SD)	0.91 (2.28)^a^	0.62 (1.88)^b^	0.34 (1.32)^c^	0.14 (0.70)^d^	0.01 (0.14)^cd^	
**FET cycle characteristics**						
**Number of FET cycles**	8999	13 510	6662	1805	268	
**Endometrium preparation protocol**						<0.001
Natural cycle	1473 (16.37%)^a^	2539 (18.79%)^b^	1431 (21.48%)^c^	394 (21.83%)^c^	60 (22.39%)^b,c^	
Hormone replacement cycle	2940 (32.67%)^a^	4593 (34.00%)^b^	2310 (34.67%)^b^	691 (38.28%)^c^	146 (54.48%)^d^	
Letrozole stimulation cycle	2921 (32.46%)^a^	3941 (29.17%)^b^	1636 (24.56%)^c^	349 (19.34%)^d^	21 (7.84%)^e^	
hMG late stimulation cycle	1655 (18.39%)^a^	2406 (17.81%)^a^	1256 (18.85%)^a^	353 (19.56%)^a^	40 (14.93%)^a^	
Missing	10 (0.11%)^a^	31 (0.23%)^b^	29 (0.44%)^c^	18 (1.00%)^d^	1 (0.37%)^a,b,c,d^	
**Stage of transferred embryos**						<0.001
Cleavage embryo transfer, n (%)	7332 (81.48%)^a^	10 806 (79.99%)^b^	5397 (81.01%)^a,b^	1541 (85.37%)^c^	255 (95.15%)^d^	
Blastocyst embryo transfer, n (%)	1651 (18.35%)^a^	2685 (19.87%)^b^	1249 (18.75 %)^a,b^	263 (14.57%)^c^	12 (4.48%)^d^	
Combined, n (%)	16 (0.18%)^a^	19 (0.14%)^a^	16 (0.24%)^a^	1 (0.06%)^a^	1 (0.37%)^a^	
**Implantation rate (%)**	66.11^a^	62.12^b^	57.30^c^	41.05^d^	14.55^e^	<0.001
**Clinical pregnancy rate (%)**	61.45^a^	57.38^b^	52.66^c^	35.84^d^	11.94^e^	<0.001
**Live birth rate (%)**	51.83^a^	47.88^b^	41.16^c^	22.83^d^	6.34^e^	<0.001

Categorical variables were tested by the chi-square test. Continuous variables were tested by one-way ANOVA. The Bonferroni correction was applied to prevent data from incorrectly appearing to be statistically significant, by making an adjustment during comparison testing. Different letters a, b, c, d, and e represent significant differences between groups. FET, frozen embryo transfer; hMG, menopausal gonadotropin; PPOS, progestin-primed ovarian stimulation; MPA, medroxyprogesterone acetate; DYG, dydrogesterone; AFC, antral follicle count.

In each age group, from youngest to oldest, the rates of ICSI insemination were 27.24%, 25.26%, 28.03%, 34.54%, and 29.38% (*P* < 0.001). AFC, an indicator of ovarian reserve, declined with increasing age. The number of oocytes retrieved, fertilized oocytes, total embryos, and high-quality embryos also became fewer with age (Table 2). The transferred embryos were primarily in the cleavage stage in all groups. However, from groups 3 to 5, there was a tendency towards an increase in the proportion of ETs at the cleavage stage. The implantation rate, clinical pregnancy rate, and live birth rate had similar trends (51.83%, 47.88%, 41.16%, 22.83%, 6.34%; *P* < 0.001), decreasing significantly with age.

However, in comparison to the previous cohort, patients undergoing non-purely PPOS protocols had inferior cycle outcomes and live birth rates, as indicated by fewer oocytes retrieved, suboptimal embryo results, and lower live birth rates (29.50%, 28.07%, 24.28%, 10.86%, and 1.85%, *P* < 0.001) (Supplementary Table S2).

### The CLBRs of different groups

**Figure 2.**
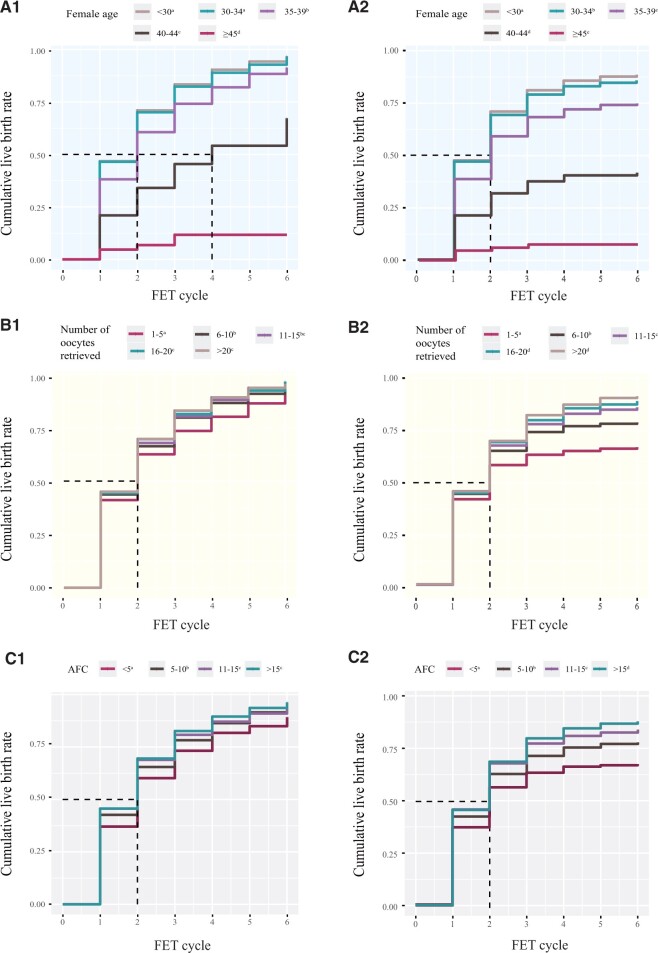
**Cumulative live birth curves for patients undergoing six frozen embryo transfer cycles, stratified by female age, number of oocytes retrieved and antral follicle count.** (**A**) The women were categorized by age (years). The CLBR was calculated by Kaplan–Meier analysis (the optimistic method) (A1) and the competing risk method (the conservative method) (A2). (**B**) The women were stratified according to the number of oocytes retrieved from their first oocyte retrieval cycle. The CLBR was calculated by Kaplan–Meier analysis (the optimistic method) (B1) and the competing risk method (the conservative method) (B2). (**C**) The women were stratified according to the AFC. The CLBR was calculated by Kaplan–Meier analysis (the optimistic method) (C1) and the competing risk method (the conservative method) (C2). The result of the pairwise comparison between the five groups is displayed by an adjusted pairwise log-rank test. *P* < 0.05 was significant. The distinct letters a, b, c, d, and e above the group legends indicate statistically significant differences between the groups. CLBR, cumulative live birth rate; FET, frozen embryo transfer; AFC, antral follicle count.

The CLBRs with the optimistic method over six FET cycles were 96.9%, 96.6%, 91.4%, 67.3%, and 11.7% for female age groups 1, 2, 3, 4, and 5, respectively (Fig. 2A1). For those treated with the conservative method (Fig. 2A2), the CLBRs in female age groups 1, 2, 3, 4, and 5, respectively, were 87.3%, 85.0%, 74.0%, 41.3%, and 7.5%. The pairwise comparisons between Group 1 and Group 2 with the optimistic method did not yield significant results (*P* = 0.148), whereas all other pairwise comparisons revealed significant differences (*P* < 0.001, Fig. 2A). The time to achieve a CLBR of 50% showed a gradual lengthening trend: Groups 1–3 achieved it by the second cycle, Group 4 achieved it by the fourth cycle, and Group 5 did not reach this in the optimistic method. By the conservative method, Groups 1–3 achieved a CLBR of 50% by the second cycle, while Groups 4–5 did not reach this.

The CLBRs with the optimistic method over six FET cycles were 93.8%, 94.3%, 95.8%, 96.0%, and 95.6% for the number of oocytes retrieved in Groups 1, 2, 3, 4 and 5, respectively (Fig. 2B1), and those with the conservative method were 66.2%, 78.3%, 85.6%, 88.9%, and 91.0% (Fig. 2B2). The pairwise comparisons between the groups are presented in Fig. 2B. The time needed to achieve a 50% CLBR for Groups 1–5 was the second cycle, by both the optimistic and conservative methods.

When the optimistic approach was used, the CLBRs over six FET cycles were 89.2%, 94.8%, 95.9%, and 96.3% for AFC Groups 1, 2, 3 and 4, respectively (Fig. 2C1). When the conservative method was used, the CLBRs over six FET cycles were 67.4%, 78.2%, 83.9%, and 88.1% for AFC Groups 1, 2, 3, and 4, respectively (Fig. 2C2). Pairwise comparisons between the groups are presented in Fig. 2C. The time needed to achieve a CLBR of 50% in Groups 1–4 was the second cycle for both the optimistic and conservative methods.

When the CLBRs of patients under non-pure PPOS-related protocols were considered, it was evident that the CLBRs were consistently lower than those in the main analysis for both the overall CLBR and the time taken to achieve a 50% CLBR. Comparing the female age groups, the rates were 94.4%, 92.1%, 81.7%, 30.8%, and 18.4% in Groups 1, 2, 3, 4, and 5, respectively, using the optimistic method and 78.6%, 72.3%, 58.1%, 19.3%, and 3.4% in Groups 1, 2, 3, 4, and 5 using the conservative method, respectively. Dividing the women by the number of oocytes retrieved, the percentages were 81.2%, 88.3%, 84.9%, 89.1%, and 84.7% with the optimistic method and 54.4%, 69.1%, 72.3%, 76.6%, and 73.8% with the conservative method. Comparing the AFC groups, the percentages were 79.7%, 84.5%, 86.3%, and 91.1% with the optimistic method and 48.3%, 63.1%, 69.3%, and 78.9% with the conservative method. Additionally, it was observed that FET cycles needed to achieve a 50% CLBR were higher by one or two cycles in the supplementary analysis (Supplementary Fig. S1).

### The impact of female age, number of oocytes retrieved, AFC, and female BMI on CLBR

**Figure 3.**
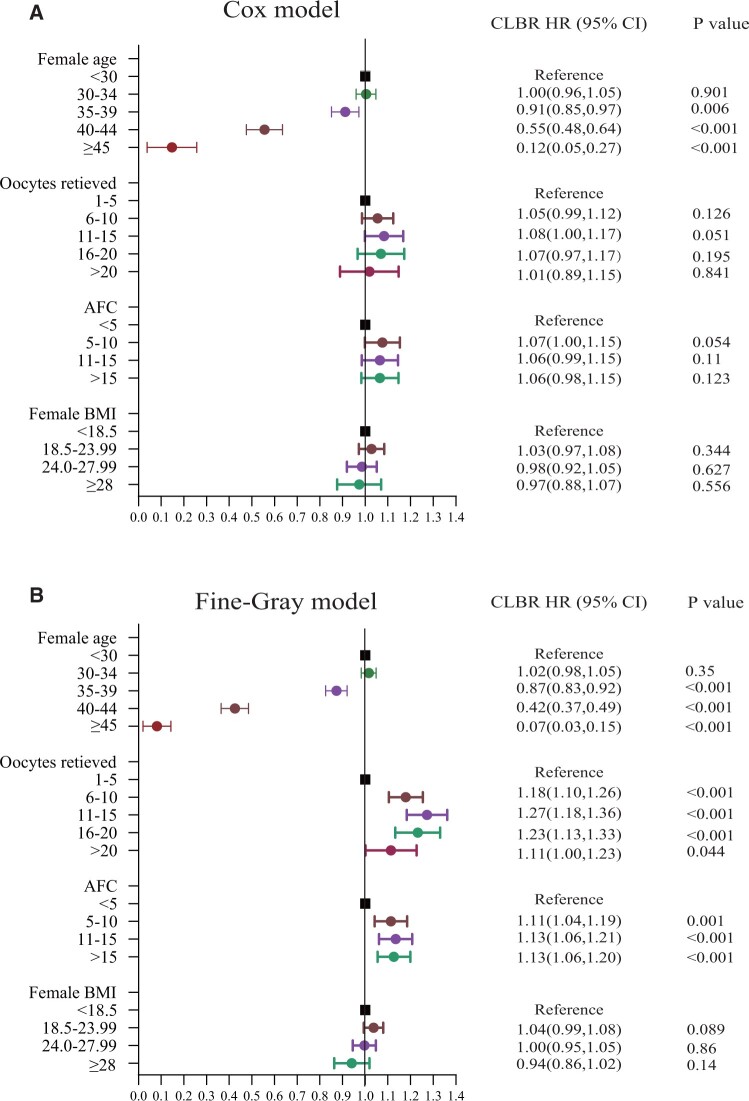
**Factors influencing the cumulative live birth rate (CLBR) within six frozen embryo transfer cycles.** The impact of female age (years), the number of oocytes retrieved, antral follicle count (AFC), and female BMI (kg/m^2^) on the CLBR was assessed using both the Cox model (**A**) and Fine–Gray model (**B**) under multivariable analysis. The following factors were adjusted for in both models: duration of infertility, male age, infertility type, infertility reason, PCOS, oocyte pick-up (OPU) number, frozen embryo transfer number, total oocyte number, total embryo number, number of embryos transferred, number of good-quality embryos transferred, and the patient’s first OPU year. Statistical significance was accepted at *P* < 0.05. The reference groups were defined as <30 years for female age, 1–5 for number of oocytes recovered, and <5 for AFC. HR, hazard ratio; CLBR, cumulative live birth rate; AFC, antral follicle count.

Univariable analysis of Cox models was used to screen potential confounding factors affecting the CLBR (Supplementary Table S3). A significance level of *P* < 0.05 was considered. The potential confounding factors, including duration of infertility, male age, and basal serum FSH value, are listed in Supplementary Table S3. Multivariable analysis was conducted using both the Cox model and the Fine–Gray model (Fig. 3) with female age, number of oocytes retrieved, AFC, female BMI, and the aforementioned potential confounding variables included. Factors that had a significant impact on CLBR after adjustment are also listed in Supplementary Table S3.

The stratification of female age demonstrated the impact of age on the CLBR (Fig. 3). When Group 1 (<30 years old) was set as the reference, there was no significant difference in the Cox and Fine–Gray models for women aged 30–34 years. However, for the group of women aged 35–39 years; 40–44 years, and ≥45 years, there was a gradual decrease in the HR. These two models further confirmed that increasing female age significantly decreased CLBR within six FET cycles but had less of an effect after age 35.

Stratification by the number of oocytes retrieved revealed an impact on CLBR (Fig. 3). When Group 1 (1–5 oocytes retrieved) was set as the reference, the groups with 6–10 oocytes retrieved had a slight increase in CLBR according to Cox and Fine–Gray regression analysis. Similarly, groups with 11–15 oocytes retrieved demonstrated a gradual increase in aHR for CLBR. However, for Group 4 and Group 5, where more than 15 oocytes were retrieved, there was no further increase in aHR observed according to the Cox regression analysis for both Group 4 and Group 5, while Fine–Gray analysis indicated that there was even a decrease in CLBR in both Group 4 and Group 5. These findings suggest that once the number of oocytes retrieved reaches 15, any additional increase has little effect on the increase in the CLBR.

The stratification of AFC revealed a significant association between AFC and CLBR (Fig. 3). When Group 1 (<5 AFC) was set as the reference, there was no significant difference in the Cox model for AFC 5–10, or AFC >15. However, there were significant differences in the Fine–Gray model for AFC 5–10, 11–15, and >15.

Stratification by BMI demonstrated the influence of BMI on CLBR (Fig. 3). When Group 1 (<18.5) was set as the reference category, there was no significant difference in Group 2 (18.5–23.99), Group 3 (24–27.99), or Group 4 (≥28) in the Cox or Fine–Gray model.

## Discussion

As the originator of the PPOS protocol, our centre has compiled the largest clinical database of patients using PPOS-related protocols. The study presented here is the first to investigate the CLBR of women with different clinical characteristics undergoing treatment with PPOS-related protocols. We studied the CLBRs of 18 593 women of varying age, number of retrieved oocytes, and AFC within six FET cycles using both optimistic and conservative methods. We utilized Cox and Fine–Gray models to analyze the impact of these three parameters on CLBR. This research, which utilizes the CLBR per woman as an overall indicator, provides a more useful reference for estimating the odds of achieving pregnancy for patients utilizing PPOS-related protocols in clinics.

Our results indicate that PPOS-related protocols can increase the CLBR by increasing the number of FETs when women are less than 45 years of age, and especially under 40 years. However, for patients over 45 years old, it is difficult to achieve a satisfactory CLBR by increasing the number of FETs. Among female patients under 40 years of age, the CLBR reached 74.0∼87.3% (conservative method) and even approached 91.4∼96.9% (optimistic method) by increasing the number of FETs to six cycles. Among female patients aged between 40 and 44 years, within six FET cycles the CLBR reached 41.3% (conservative method) and 67.3% (optimistic method). The CLBR tended to decline with increasing age, and the most significant decline occurred after the age of 45 years, which is consistent with a previous study (Devesa *et al.*, 2018). However, in our study, the CLBRs of older patients were slightly higher than those reported in another study of PPOS-related protocols. In our research, the CLBRs for patients aged 35–39 years were 74.0% and 91.4% in the conservative and optimistic methods, respectively, while another study utilizing a mild stimulation protocol reported rates of only 9% and 10% (Tu *et al.*, 2021). For women aged 36–39 years using the long protocol, the CLBR ranged from 26.7% in double-ET to 41.8% in single-ET (Veleva *et al*., 2006), whereas for women aged 40–44 years using either the GnRH-agonist or antagonist regimen, it varied between 13.2% and 22.7% (Niinimäki *et al.*, 2013). Furthermore, protocols such as the GnRH-agonist microdose flare protocol, mid-luteal GnRH agonist long protocol, or GnRH antagonist short protocol led to a CLBR of approximately 31.1% and 28.0% for women aged between 40 and 43 years (Tannus *et al.*, 2017). The study that best aligns with our reported CLBRs demonstrated that women aged between 35 and 40 years achieved a CLBR of approximately 51–66% in double-ET and 56–70% in single-ET, without specifying the stimulation protocol (Mejia *et al.*, 2021). PPOS-related protocols are preferable for older women or those with low ovarian reserve because they are better at preventing early LH surges than GnRH antagonists and thus help in obtaining more mature oocytes (Hossein Rashidi *et al.*, 2020). Perhaps for this reason, the PPOS-related scheme has an advantage over the traditional scheme in women above 40 years old.

When more than five oocytes were retrieved, the CLBR reached 94.3% (optimistic method) or 78.3% (conservative method) and even reached 95.6% (optimistic method) or 91.0% (conservative method) in certain groups. Even when fewer than five oocytes were collected, the time to achieve 50% CLBR was not delayed (second cycle) compared to the time in the other groups for both the optimistic and conservative methods. A previous study with a GnRH antagonist ovulatory stimulation protocol showed that the CLBR could reach 90% in patients’ first ‘freeze-all’ cycles (IVF/ICSI) when the number of oocytes retrieved was more than 10 (Zhao *et al.*, 2020). This figure is slightly higher (85.6%) than our corresponding calculation method (the conservative method), which could, in part, be because their study included only women younger than 35 years old. Li *et al.* (2019) showed a relatively low CLBR (14.6% for the <10 oocytes group, 33.2% for the 10–15 group, and 56.8% for the >15 group) of the ‘freeze-all’ strategy, stratified by the number of oocytes retrieved, compared with our research. Law *et al.* (2019) demonstrated a positive correlation between the CLBR per aspiration and the number of oocytes retrieved in both fresh and freeze-all strategies. Specifically, Groups 1–3 had a CLBR of 10.0%, the group with 4–9 oocytes had a CLBR of 27.6%, groups 10–14 had a CLBR of 44.1%, groups 15–19 had a CLBR of 53.7%, groups 20–24 had a CLBR of 60.4%, and the ≥25 oocyte retrieval group reached 66.2%. Polyzos *et al.* (2018) also saw a gradual increase in CLBR with the number of retrieved oocytes, achieving rates up to 70% when ≥25 oocytes were retrieved. While there was some variation in their results, their CLBR outcomes were similar or slightly lower than the results of our study. The different ovarian stimulation programmes, which could result in varying numbers of oocytes retrieved, and different FET cycles are also probable reasons for these differences. PPOS-related protocols effectively prevent premature LH surges while achieving equivalent outcomes in terms of oocyte retrieval and pregnancy rates (Kuang *et al.*, 2015). This factor could explain why groups with few retrieved oocytes using the PPOS-related strategy had a higher CLBR.

Compared with female age, AFC had less influence on the CLBR of patients when applying the PPOS-related scheme. Although the CLBRs of the groups with AFC < 10 were relatively low compared with those of the other groups, the CLBR continued to increase continuously with more FETs. However, the time to achieve a 50% CLBR was the same in all four groups (in the second cycle). AFC is an indicator of ovarian reserve. In our research, there was a decrease in the percentage of women older than 40 years as the number of AFCs increased (data not shown). Furthermore, the AFC 1–5 group had the highest proportion of advanced female age; hence, it can be inferred that the good result seen among low AFC counts (1–5 and 6–10 groups) is not solely attributed to a lower representation of older individuals. Previous studies have produced mixed results regarding the efficacy of the PPOS strategy in patients with a low ovarian reserve. The CLBR is significantly higher with a GnRH antagonist than with PPOS in low-prognosis patients, as found in a previous study (Zhang *et al.*, 2021). However, the mainstream opinion in this field is that for patients with a low ovarian reserve, the PPOS strategy has an equivalent or even better effect on pregnancy outcomes than does the GnRH-ant protocol (Huang *et al.*, 2019; Guan *et al.*, 2021) or mild stimulation protocol (Tu *et al.*, 2021). Furthermore, some of these studies (Guan *et al.*, 2021) also showed that more oocytes and top-quality embryos were obtained in the PPOS group, which positively correlated with the CLBR, than with the mild stimulation protocol. The current research further supports the advantage of using the PPOS strategy for patients with poor ovarian reserve. In fact, over six FET cycles, the CLBR reached 89.2% (optimistic method) or 67.4% (conservative method) among individuals in the low-AFC group (<5).

The results of the multivariable analysis (Fig. 3) suggest that advanced age, few AFCs, and high BMI have an interactive influence on CLBR. Among women with low AFC and high BMI, advanced age emerges as the primary factor impacting CLBR. For instance, while AFC < 5 or BMI ≥ 28 kg/m^2^ may have a lesser impact on CLBR when using the PPOS-related protocol, the data in Fig. 2A and B indicate that an advanced age of 40–44 years might restrict CLBR by up to 50%. We also included patients who underwent treatment with non-pure PPOS-related protocols in our supplementary analysis. Compared to those who underwent purely PPOS-related protocols, this group of patients who underwent non-purely PPOS-related protocols had distinct characteristics, including a higher proportion of older patients, more OPU cycles, more FET cycles, and inferior pregnancy outcomes (Supplementary Tables S1 and S2 and Fig. S1). Patients utilizing purely PPOS-related protocols needed fewer FET cycles to achieve a 50% CLBR and had higher ultimate CLBRs within FET cycles in all stratified groups (Fig. 2 and Supplementary Fig. S1). Despite the differences in basic characteristics between these two patient groups, the discrepancy in CLBR suggests that using purely PPOS-related protocols may lead to better pregnancy outcomes than using non-purely PPOS-related protocols.

The current research is limited by its single-centre and retrospective nature; however, the large population size with different female ages, numbers of oocytes retrieved, and AFCs, along with the indicator of ‘CLBR per woman,’ can provide a valuable reference for patients worldwide who adopt the PPOS-related scheme. Additionally, the high number of drop-outs (which increases with increasing FET number) is an inevitable limitation that can be minimized by large population studies. The predictive nature of the CLBR is determined by analyzing both the optimistic and conservative methods and accounting for existing patients who have remaining embryos but choose not to proceed with FET. Therefore, the actual CLBR curve falls within a range between optimism and pessimism.

In conclusion, this study reports the CLBR per woman rather than per cycle to provide more representative results, reflecting real-life scenarios. Furthermore, conducting such a comprehensive analysis using a large-scale dataset encompassing significant numbers of reported live births offers patients utilizing the PPOS-related strategy in clinics invaluable reference points for choosing whether to continue ART treatment after unsuccessful transfers. Our findings suggest that women below 45 years old, especially younger than 40 years, can significantly increase their chances of achieving a successful live delivery by undergoing additional FETs. Women over 45 years do not benefit from this approach. Furthermore, women with lower AFCs and fewer retrieved oocytes may consider pursuing the PPOS-related scheme and repeating FETs to improve their CLBR outcomes.

## Supplementary Material

hoad051_Supplementary_Tables_final

hoad051_Supplementary_Figure_S120230926

## Data Availability

The data underlying this article will be shared upon reasonable request to the corresponding author.
