# Supplementary material for: The cumulative live birth rates of 18 593 women with progestin-primed ovarian stimulation-related protocols and frozen-thawed transfer cycles
Source: Hum Reprod Open. 2023 Dec 21;2024(1):hoad051. doi: 10.1093/hropen/hoad051 (PMC10769816; doi:10.1093/hropen/hoad051)
Supplement: hoad051_Supplementary_Tables_final [file hoad051_supplementary_tables_final.docx]

**Supplementary Table S1:** Baseline characteristics of 8763 female patients under non-pure PPOS-related protocols.

| **Demographic Characteristics** | **N (%)** |
| --- | --- |
| **Number of women** | 8763 |
| **Female age (y)** |  |
| <30 | 1697 (19.40%) |
| 30-34 | 3040 (34.69%) |
| 35-39 | 2260 (25.79%) |
| 40-44 | 1438 (16.41%) |
| ≥45 | 328 (3.74%) |
| **Female BMI (kg/m^2^)** |  |
| <18.5 | 839 (9.57%) |
| 18.5-23.99 | 5886 (67.17%) |
| 24.0-27.99 | 1614 (18.42%) |
| ≥28.0 | 424 (4.84%) |
| **Infertility type** |  |
| Primary infertility | 4406 (50.28%) |
| Secondary infertility | 4357 (49.72%) |
| **Cause of infertility** |  |
| Tubal | 3335 (38.06%) |
| Male cause | 740 (8.44%) |
| Ovulatory | 693 (7.91%) |
| Endometrium factor | 392 (4.47%) |
| Mixed causes | 2825 (32.24%) |
| Unexplained | 778 (8.88%) |
| **Duration of infertility (y)** | 3.79 |
| **Number of oocyte pick-up cycles** | 33 704 |
| IVF | 18 498 (54.88%) |
| ICSI | 13 021 (38.63%) |
| IVF+ICSI | 729 (2.16%) |
| Missing | 1456 (4.32%) |
| **Number of women having different**  **number of oocyte pick-up cycles** |  |
| 1 | 0 |
| 2 | 3276 (37.38%) |
| 3 | 1978 (22.57%) |
| 4 | 1266 (14.45%) |
| ≥5 | 2243 (25.60%) |
| **Number of FET cycles** | 19 627 |
| **Number of women having different FET cycles and mean duration (month)** |  |
| 1 | 2837 (32.37%); 7.33 |
| 2 | 2387 (27.24%); 14.25 |
| 3 | 1521 (17.36%); 21.85 |
| 4 | 775 (8.84%); 28.63 |
| 5 | 374 (4.27%); 35.84 |
| ≥6 | 358 (4.09%); 43.99 |
| People with no FET after OPU | 511 (5.83%) |

OPU: oocyte pick-up FET: frozen embryo transfer, PPOS: progestin-primed ovarian stimulation

**Supplementary Table S2:** Characteristics of IVF/ICSI and FET cycles in female patients of different age groups under non-pure PPOS-related COH protocols. **­­­**

| **­­­IVF/ICSI cycle characteristics** | | | | | | |
| --- | --- | --- | --- | --- | --- | --- |
|  | **<30** | **30-34** | **35-39** | **40-44** | **≥45** | **P value** |
| **Number of oocyte pick-up cycles** | 4186 | 9776 | 9388 | 7634 | 2720 | <0.001 |
| IVF | 2409 (57.55%)^a^ | 5602 (57.30%)^a^ | 5158 (54.94%)^b^ | 3984 (52.19%)^c^ | 1345 (49.45%)^c^ |  |
| ICSI | 1486 (35.50%)^a^ | 3553 (36.34%)^a^ | 3697 (39.38%)^b^ | 3114 (40.79%)^bc^ | 1171 (43.05%)^c^ |  |
| IVF+ICSI | 198 (4.73%)^a^ | 350 (3.58%)^b^ | 131 (1.40%)^c^ | 45 (0.59%)^d^ | 5 (0.18%)^d^ |  |
| Missing | 93 (2.22%)^a^ | 271 (2.77%)^a^ | 402 (4.28%)^b^ | 491 (6.43%)^c^ | 199 (7.32%)^c^ |  |
| **COH protocol** |  |  |  |  |  | <0.001 |
| PPOS related protocols | 3450 (82.42%)^ab^ | 8118 (83.04%)^b^ | 7625 (81.22%)^a^ | 6155 (80.63%)^a^ | 2120 (77.94%)^c^ |  |
| Short protocol | 304 (7.26%)^a^ | 634 (6.49%)^a^ | 398 (4.24%)^b^ | 156 (2.04%)^c^ | 27 (0.99%)^d^ |  |
| Long protocol | 75 (1.79%)^a^ | 152 (1.55%)^a^ | 130 (1.38%)^a^ | 58 (0.76%)^b^ | 6 (0.22%)^c^ |  |
| GnRH antagonist protocol | 158 (3.77%)^a^ | 336 (3.44%)^a^ | 393 (4.19%)^a^ | 274 (3.59%)^a^ | 93 (3.42%)^a^ |  |
| Other | 116 (2.77%)^a^ | 289 (2.96%)^a^ | 415 (4.42%)^b^ | 498 (6.52%)^c^ | 201 (7.39%)^c^ |  |
| Missing | 83 (1.98%)^a^ | 247 (2.53%)^a^ | 427 (4.55%)^b^ | 493 (6.46%)^c^ | 273 (10.04%)^d^ |  |
| **AFC, mean (SD)** | 9.93 (8.19)^a^ | 7.24 (6.61)^b^ | 5.11 (4.87)^c^ | 3.51 (3.35)^d^ | 2.52 (2.55)^e^ | <0.001 |
| **Oocytes retrieved, mean (SD)** | 7.44 (6.92)^a^ | 5.46 (5.53)^b^ | 3.73 (3.99)^c^ | 2.36 (2.55)^d^ | 1.69 (1.75)^e^ | <0.001 |
| **Fertilized oocytes, mean (SD)** | 4.94 (4.79)^a^ | 3.86 (3.99)^b^ | 2.73 (2.98)^c^ | 1.8 (1.99)^d^ | 1.32 (1.44)^e^ | <0.001 |
| **Total embryos, mean (SD)** | 4.26 (4.35)^a^ | 3.33 (3.64)^b^ | 2.34 (2.72)^c^ | 1.51 (1.83)^d^ | 1.07 (1.31)^e^ | <0.001 |
| **High-quality embryos** |  |  |  |  |  | <0.001 |
| Cleavage embryos, mean (SD) | 1.57 (1.81)^a^ | 1.33 (1.61)^b^ | 1.1 (1.35)^c^ | 0.83 (1.06)^d^ | 0.66 (0.89)^e^ |  |
| Blastocyst embryos, mean (SD) | 0.12 (0.82)^a^ | 0.09 (0.68)^b^ | 0.05 (0.48)^c^ | 0.01 (0.19)^d^ | 0.01 (0.08)^e^ |  |
| **FET cycle characteristics** | | | | | | |
|  | **<30** | **30-34** | **35-39** | **40-44** | **≥45** | **P value** |
| **Number of FET cycles** | 2671 | 6460 | 5448 | 3700 | 1348 |  |
| **Endometrium Preparation Protocol** |  |  |  |  |  | <0.001 |
| Natural cycle | 514 (19.24%)^a^ | 1371 (21.22%)^ab^ | 1250 (22.94%)^b^ | 769 (20.78%)^ab^ | 259 (19.21%)^a^ |  |
| Hormone replacement cycle | 971 (36.35%)^a^ | 2471 (38.25%)^a^ | 2289 (42.02%)^b^ | 1832 (49.51%)^c^ | 799 (59.27%)^d^ |  |
| Letrozole stimulation cycle | 696 (26.06%)^a^ | 1437 (22.24%)^b^ | 977 (17.93%)^c^ | 473 (12.78%)^d^ | 133 (9.87%)^e^ |  |
| HMG late stimulation cycle | 484 (18.12%)^a^ | 1170 (18.11%)^a^ | 922 (16.92%)^a^ | 620 (16.76%)^a^ | 154 (11.42%)^b^ |  |
| Missing | 6 (0.22%)^a^ | 11 (0.17%)^a^ | 10 (0.18%)^a^ | 6 (0.16%)^a^ | 3 (0.22%)^a^ |  |
| **Stage of transferred embryos** |  |  |  |  |  | <0.001 |
| Cleavage embryo transfer, n (%) | 2221 (83.15%)^ab^ | 5319 (82.34%)^b^ | 4626 (84.91%)^a^ | 3307 (89.38%)^c^ | 1293 (95.92%)^d^ |  |
| Blastocyst embryo transfer, n (%) | 440 (16.47%)^ab^ | 1124 (17.40%)^b^ | 798 (14.65%)^a^ | 380 (10.27%)^c^ | 52 (3.86%)^d^ |  |
| Combined, n (%) | 10 (0.37%)^a^ | 17 (0.26%)^a^ | 24 (0.44%)^a^ | 13 (0.35%)^a^ | 3 (0.22%)^a^ |  |
| **Implantation rate (%)** | 44.37^a^ | 43.42^a^ | 40.12^b^ | 26.32^c^ | 9.35^d^ | <0.001 |
| **Clinical pregnancy rate (%)** | 39.12^a^ | 37.76^a^ | 35.15^b^ | 21.51^c^ | 6.23^d^ | <0.001 |
| **Live birth rate (%)** | 29.50 ^a^ | 28.07^a^ | 24.28^b^ | 10.86^c^ | 1.85^d^ | <0.001 |

Categorical variables were tested by the chi-square test. Continuous variables were tested by one-way ANOVA. The Bonferroni correction was applied to prevent data from incorrectly appearing to be statistically significant, by making an adjustment during comparison testing. Different letters a, b, c, d and e represent significant differences between groups. PPOS: progestin-primed ovarian stimulation MPA: medroxyprogesterone acetate, DYG: dydrogesterone, AFC: antral follicle count, SD: standard deviation, FET: frozen embryo transfer, COH: controlled ovarian hyperstimulation.

**Supplementary Table S3:** The single-variable and multivariable analysis of Cox models of potential confounding factors affecting the CLBR of patients under pure PPOS-related protocol.

| **Confounding factors** | **HR (95%Ci)** | **P value** | **Adjusted HR (95%Ci)** | **P value** |
| --- | --- | --- | --- | --- |
| **Duration of infertility** | 0.98 (0.97,0.99) | <0.001 | 0.99 (0.98,1.00) | 0.003 |
| **Male age** |  | <0.001 |  | 0.383 |
| <30 | Reference |  | Reference |  |
| 30-34 | 1.25 (1.1,1.43) | 0.001 | 1.12 (0.98,1.28) | 0.085 |
| 35-39 | 1.27 (1.12,1.45) | <0.001 | 1.12 (0.98,1.28) | 0.095 |
| 40-44 | 1.19 (1.05,1.36) | 0.007 | 1.12 (0.97,1.29) | 0.115 |
| ≥45 | 0.97 (0.85,1.11) | 0.66 | 1.09 (0.93,1.26) | 0.284 |
| **Infertility type** |  |  |  |  |
| Primary infertility | Reference |  | Reference |  |
| Secondary infertility | 0.89 (0.86,0.92) | <0.001 | 0.95 (0.91,0.98) | 0.002 |
| **Infertility reason** |  | 0.012 |  | 0.666 |
| Tubal | Reference |  |  |  |
| Male cause | 1.05 (1.00,1.11) | 0.058 | 1.00 (0.95,1.06) | 0.985 |
| Ovulatory | 0.95 (0.88,1.03) | 0.197 | 0.96 (0.87,1.05) | 0.379 |
| Endometrium factor | 0.94 (0.85,1.04) | 0.243 | 0.96 (0.87,1.06) | 0.442 |
| Mixed causes | 0.95 (0.91,1.00) | 0.027 | 0.96 (0.92,1.01) | 0.107 |
| Unexplained | 1.01 (0.95,1.07) | 0.765 | 0.99 (0.94,1.06) | 0.843 |
| **PCOS** |  |  |  |  |
| No | Reference |  | Reference |  |
| Yes | 1.07 (1.01,1.12) | 0.017 | 1.04 (0.97,1.12) | 0.31 |
| **OPU times** | 0.65 (0.62,0.68) | <0.001 | 0.95 (0.90,1.00) | 0.072 |
| **FET times** | 0.01 (0.00,0.01) | <0.001 | 0.00 (0.00,0.01) | <0.001 |
| **Total oocyte number** | 0.99 (0.99,0.99) | <0.001 | 1.00 (0.99,1.01) | 0.92 |
| **Total embryo number** | 0.99 (0.98,0.99) | <0.001 | 1.01 (1.01,1.02) | <0.001 |
| **Number of embryos transferred** | 0.54 (0.53,0.54) | <0.001 | 1.09 (1.06,1.13) | <0.001 |
| **Number of good-quality embryos transferred** | 0.79 (0.77,0.81) | <0.001 | 1.02 (0.99,1.05) | 0.145 |
| **Patients’ first OPU year** |  | <0.001 |  |  |
| 2011-2013 | Reference |  | Reference |  |
| 2014-2015 | 1.03 (0.93,1.13) | 0.58 | 1.05 (0.95,1.16) | 0.356 |
| 2016-2017 | 1.08 (0.98,1.19) | 0.107 | 1.02 (0.92,1.13) | 0.728 |
| 2018-2019 | 1.10 (0.99,1.21) | 0.067 | 1.05 (0.94,1.17) | 0.4 |
| 2020-2022 | 0.72 (0.65,0.81) | <0.001 | 0.73 (0.64,0.82) | <0.001 |

CLBR: cumulative live birth rate, HR: hazard ratio, OPU: oocyte pick-up, FET: frozen embryo transfer, PPOS: progestin-primed ovarian stimulation.
