# Supplementary material for: The cumulative live birth rates of 18 593 women with progestin-primed ovarian stimulation-related protocols and frozen-thawed transfer cycles
Source: Hum Reprod Open. 2023 Dec 21;2024(1):hoad051. doi: 10.1093/hropen/hoad051 (PMC10769816; doi:10.1093/hropen/hoad051)
Supplement: hoad051_Supplementary_Figure_S120230926 [file hoad051_supplementary_figure_s120230926.pdf]

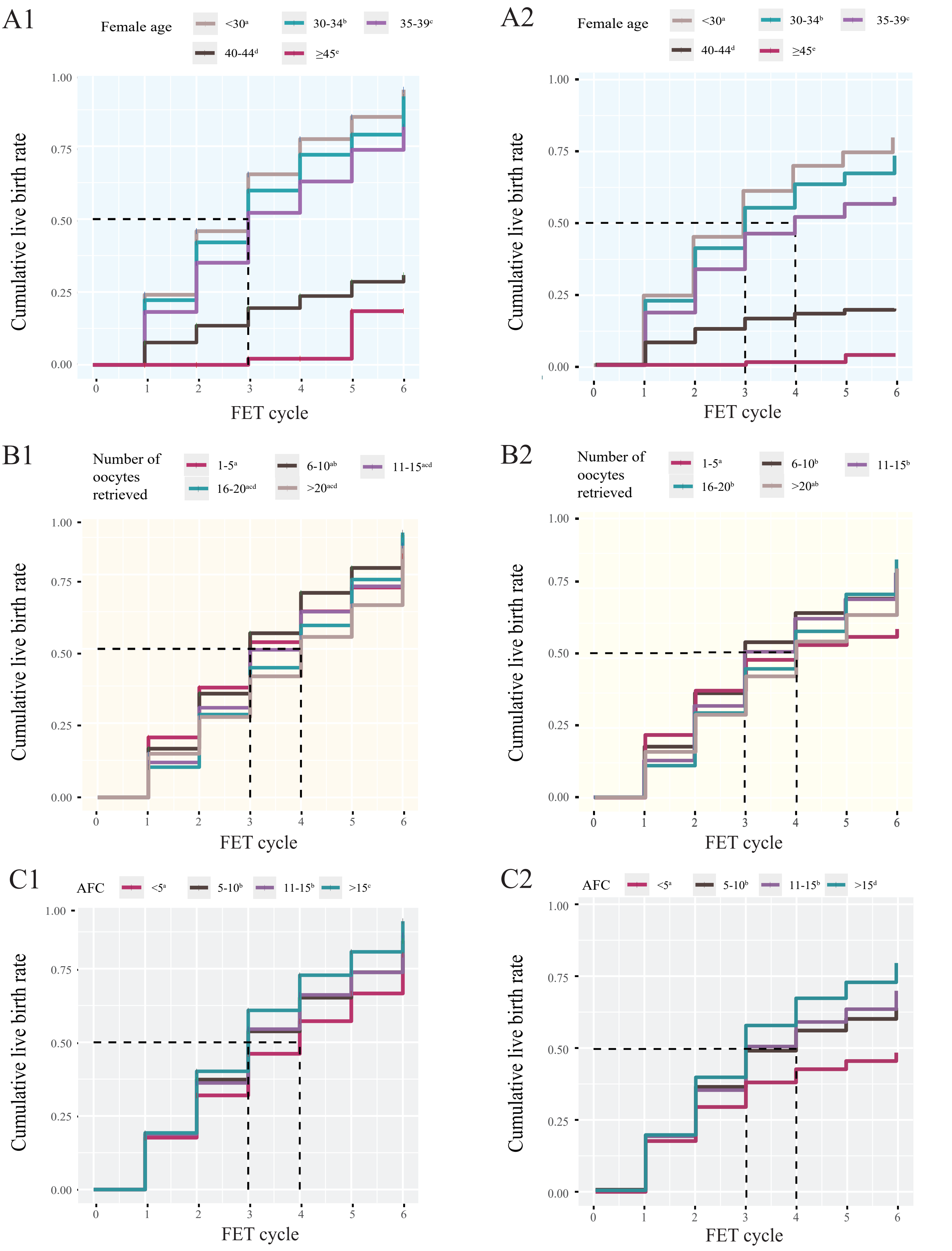

Supplementary Figure S1: Cumulative live birth curves for patients undergoing non-pure PPOS-related protocols, categorized by female age, number of oocytes retrieved, and AFC. (A): Female patients were categorized based on their age. The cumulative live birth rate was calculated by Kaplan-Meier analysis (the optimistic method) (A1) and the competing risk method (the conservative method) (A2). (B): Female patients were stratified according to the number of oocytes retrieved of their first oocyte pick-up cycle. The cumulative live birth rate was calculated by Kaplan-Meier analysis (the optimistic method) (B1) and the competing risk method (the conservative method) (B2). (C) Female patients were stratified according to the AFC. The cumulative live birth rate was calculated by Kaplan-Meier analysis (the optimistic method) (C1) and the competing risk method (the conservative method) (C2). The result of the pair-wise comparison among the five groups is presented on the sign. P<0.05 was regarded as statistically significant. The distinct letters a, b, c, d and e above the group legends indicate statistically significant differences between the groups. FET: frozen embryo transfer. AFC: antral follicle count.
